# A Naughty Case

**Published:** 1872-02

**Authors:** S. J. Cobb


### ARTICLE IV.

### *A Naughty Case.*

BY DR. S. J. COBB.

Now and then we find one who has courage and manliness enough to speak of his failures. We are very much obliged to Prof. Cutler, of New Orleans, for his report of an ugly case of hemorrhage. It is said that truth is mighty and will prevail. The doctor must have had an eye single to the truthfulness of the saying when he made his report. I have asked myself frequently since reading the account of his "unpardonable blunder" as he styles it, how many of us would acknowledge it as manfully as he has done? It is nothing more than common for us to speak of our success, but very uncommon to make known our miserable failures. If the truth has to prevail, why not let it do so at once? We are of the opinion, the sooner the better.

While we have made many failures, and unpardonable at that, we are glad to say we have thus far been saved from pulling two teeth instead of one. It may be that we will do it and even worse, when we get to using nitrous oxide gas; but as we prefer our own, we think it will be sometime before such a thing occurs. Now doctor, that you have found your error, we would advise you to put your foot down and stand upon it. You say you believe gas to be a great humbug, one of the greatest evils as far as dentistry is concerned, and that multiplied thousands of teeth are annually sacrificed by its use that might be saved. If gas and other anæsthetics are not only useless, but a curse to our profession, why not abandon them?

We are glad to hear some of our leading spirits speak out against these things, but we are sorry to say, thus far, it remains for their faith to be proven by their works. We have long since satisfied ourself that there was too much risk in the administration of any general anæsthetics, to encourage its use in our profession. If as a consequence the use of these things for the purpose of extracting teeth is not humbuggery, it is the next thing to it, and as such we do not propose to do it; we will say once and for always, we never intend to convert our office into a laughing gas establishment. We did not in times past think these laughing gas dentists the best in the world, and we are glad to say time has not changed our opinion, notwithstanding some of our first-class men have reluctantly taken hold of it, and occasionally use it, to the injury of their patrons, not only by pulling out the wrong tooth once in awhile, but crazing their minds, and sometimes sending them to their long homes. Again, you say gas and cheap rubber plates are the order of the day, and no one can stop the tide. Let us see about that; suppose you and every body else that thinks as you do, that gas is a humbug, or at least unnecessary in our profession, should exert their manhood in trying to stop the evil, as you did in reporting your ugly failure; don't you suppose we would finally be able to revoke or countermand the order, and stop the tide? If you don't we do, and have made up our minds to make the effort. Is it not our duty to do truth, regardless of popular opinion? We must remember that the masses are not great investigators, as such we cannot see how truth is to assert itself, except the few who are able to find it out, stick to it. Let us have truth, and it will give us what Mr. Grant used to call for, "peace."

---

